# A Multi-Layer Intrusion Detection System for SOME/IP-Based In-Vehicle Network

**DOI:** 10.3390/s23094376

**Published:** 2023-04-28

**Authors:** Feng Luo, Zhenyu Yang, Zhaojing Zhang, Zitong Wang, Bowen Wang, Mingzhi Wu

**Affiliations:** 1 School of Automotive Studies, Tongji University, Shanghai 201804, China; luo_feng@tongji.edu.cn (F.L.); zhangzhaojing@tongji.edu.cn (Z.Z.); 1911048@tongji.edu.cn (Z.W.); bowen@tongji.edu.cn (B.W.); 2 Nanchang Automotive Institute of Intelligence and New Energy, Tongji University (NAIT), Nanchang 330052, China; wumingzhi@naiine.com

**Keywords:** intrusion detection system, SOME/IP, deep learning

## Abstract

The automotive Ethernet is gradually replacing the traditional controller area network (CAN) as the backbone network of the vehicle. As an essential protocol to solve service-based communication, Scalable service-Oriented MiddlewarE over IP (SOME/IP) is expected to be applied to an in-vehicle network (IVN). The increasing number of external attack interfaces and the protocol’s vulnerability makes SOME/IP in-vehicle networks vulnerable to intrusion. This paper proposes a multi-layer intrusion detection system (IDS) architecture, including rule-based and artificial intelligence (AI)-based modules. The rule-based module is used to detect the SOME/IP header, SOME/IP-SD message, message interval, and communication process. The AI-based module acts on the payload. We propose a SOME/IP dataset establishment method to evaluate the performance of the proposed multi-layer IDS. Experiments are carried out on a Jetson Xavier NX, showing that the accuracy of AI-based detection reached 99.7761% and that of rule-based detection was 100%. The average detection time per packet is 0.3958 ms with graphics processing unit (GPU) acceleration and 0.6669 ms with only a central processing unit (CPU). After vehicle-level real-time analyses, the proposed IDS can be deployed for distributed or select critical advanced driving assistance system (ADAS) traffic for detection in a centralized layout.

## 1. Introduction

With the continuous evolution of the Internet of Things (IoT), the vehicle has become an indispensable part [1]. The trend of IoT leads to the introduction of information technology (IT), software-defined networking (SDN) [2], and service-oriented architectural design concepts, which give automotive applications great flexibility to deploy, update, and expand the introduction of information technology (IT), and service-based architectural design concepts give automotive applications great flexibility to deploy, update and expand [3]. A large amount of external data enters the IVN through wireless technologies, such as Wi-Fi, Bluetooth, ZigBee, dedicated short-range communication (DSRC), and long-term evolution (LTE). Diverse upper-layer applications, such as safety-related, entertainment-related, and control-related applpications [4], also put forward new requirements for the backbone of the IVN. In addition to high speed and high bandwidth, the in-vehicle network also needs to be redundant, scalable, real-time, deterministic, and secure, which cannot be provided by traditional in-vehicle buses such as CAN, local interconnect network (LIN) and media-oriented system transport (MOST). The automotive Ethernet (AE) solves the electromagnetic compatibility problem using traditional Ethernet in the vehicle environment [5]. The above requirements can be satisfied by optimizing and multiplexing the protocols of different layers in the OSI model to the AE [6]. BMW proposed the SOME/IP protocol in 2011 as a critical protocol for solving service-oriented communication and was incorporated into the AUTomotive Open System Architecture (AUTOSAR) specification in 2014. Kreissl [7] obtained the vulnerability of SOME/IP through threat analysis, and some studies have proven that the in-vehicle network can be hacked through external interfaces (Bluetooth, WIFI), operating system vulnerabilities, or malware [8,9,10,11]. It is evident that SOME/IP has security risks and needs corresponding security measures. 

However, there are still some problems in deploying security countermeasures on SOME/IP. First, there is no definition of its security mechanism in AUTOSAR and no standards to guide the deployment of security measures. Second, existing security protocols, such as transport layer security (TLS) and Ipsec, do not fit well in the SOME/IP protocol. Although some papers have studied security protocols specially designed for SOME/IP [12,13,14], forming a standardized module and trade-off between encryption strength and real-time performance is difficult. Third, IDS is also an effective means to detect attacks or network anomalies [15]. Tobias et al. [16] also believe that IDS for SOME/IP has opportunities and challenges. Nevertheless, few studies on the IDS for SOME/IP-based AE exist.

The motivations of this work are as follows.

Firstly, different wireless technologies are integrated into cars, which can be an avenue for external attacks. Once the SOME/IP-based in-vehicle network is compromised, the attacker can not only affect the Internet of Vehicles (IoV) application by obfuscating the in-vehicle data but can also directly operate the actuator to cause serious accidents. Therefore, diversified security methods need to be applied to defend against different attacks. In addition, although the security countermeasures based on cryptography can guarantee the confidentiality, integrity, and availability of data, they cannot identify abnormal behaviors in the communication network without attack intervention, such as abnormal traffic caused by sensor failure or administrator misoperation. The in-vehicle network requires IDS for more comprehensive network monitoring and abnormal location. Lastly, few studies and public datasets on the IDS for SOME/IP-based AE exist. This gap urgently needs to be filled.

Based on the above considerations, this paper introduces the attack scenario of SOME/IP-based AE and analyzes the attack on the SOME/IP protocol. An innovative multi-layer intrusion detection system is proposed, incorporating both rule-based and AI-based detection methods. The establishment of the rule set mainly relies on the attack analysis results. AI detection mainly includes data pre-processing, a novel multi-gated recurrent unit (multi-GRU) model, and a Bayesian optimization process. Finally, we implement the proposed IDS and comprehensively evaluate its performance.

The main contributions of this paper are as follows:

- We propose SOME/IP data generation methods based on Prescan, Simulink, and CANoe. In addition to the SOME/IP header that satisfies the protocol specification, the method can generate meaningful and relevant in-vehicle network data, such as camera data, ADAS data, body data, and attack data.
- We propose a multi-layer intrusion detection system architecture with both rule-based and AI-based approaches. This is the first attempt to detect anomalies simultaneously on SOME/IP header, SOME/IP-SD message, message interval, and payload.
- The multi-GRU model is proposed in the AI-based method, and the detection performance is improved by data pre-processing and Bayesian optimization. Multi-GRU is shown to scale well and outperform the single-GRU model.
- We implement the IDS proposed in this paper on a laptop and Jetson Xavier NX and evaluate its performance using a simulation database. Experiments show that our proposed IDS has excellent detection accuracy and meets the real-time performance of vehicles.

The paper is organized as follows. Section 2 discusses the related work. Section 3 presents the vulnerability of SOME/IP. In Section 4, the proposed multi-Layer IDS is introduced, mainly including rule-based and AI-based modules. Section 5 presents the result of the experiment and discusses the vehicle’s real-time performance. Section 6 is the conclusion of the paper.

## 2. Related Work

There are many classification methods for intrusion detection [17,18]. From the technique of judging features, intrusion detection systems can be divided into rule-based and AI-based. Both of them need to abstract the features used for detection first. These features can be the voltage signal of the controller, the information entropy of the sampled data, the message ID and the message interval, and so on. In rule-based detection, rules are set through human observation of features and network behavior. The rule set is fixed and relies heavily on expert experience. Rule-based IDS is more suitable for static network communication. It is difficult to enumerate all the different attacks, but it often has advantages in computing performance. AI-based detection uses machine learning (ML) or deep learning (DL) techniques to learn features. The training and detection effects are closely related to the comprehensiveness of the data and the characteristics of the model. AI-based IDS has obvious advantages in large-scale and flexible networks, such as SDN [19,20,21] and IoT [22,23], but it will be resource-constrained in embedded environments.

### 2.1. IDS on CAN

NORAS et al. [24] proposed a rule-based IDS. The rules are established by observing the CAN SPEC file, including the speed increment and the message interval. Wonsuk et al. [25] used two models, the support vector machine (SVM) and bagged decision trees (BDT), to learn the time–domain and frequency–domain features of CAN electrical signals to detect anomalies. MARKUS et al. [26] designed an unsupervised signal prediction structure fused with multiple long short-term memory (LSTM) models to detect attacks by comparing the predicted signal with the actual signal. Song et al. [27] demonstrated the feasibility of a convolutional neural network (CNN) in CAN network intrusion detection. The author proposes the reduced Inception ResNet model for real-time consideration and compares the performance with LSTM, artificial neural networks (ANN), k-nearest neighbors (kNN), SVM, Decision Tree (DT), and Naive Bayes. Olufowobi et al. [28] proposed an AI-based IDS for CAN named SAIDuCANT. The author proposes a supervised learning algorithm to learn and classify the response time of each message. Yang et al. [29] proposed a multi-tiered hybrid IDS framework, including data pre-processing, feature engineering, ML-based methods (DT, Random Forest (RF), Extra Trees (ET), Extreme Gradient Boosting (XGBoost), and Cluster Labeling (CL) k-means), and model optimization. Since this is not the focus of our work, we only introduce some typical studies. There are more related works in the literature [30,31,32,33,34,35,36,37,38,39].

### 2.2. IDS on Automotive Ethernet

#### 2.2.1. Rule-Based IDS

Nadine et al. [40] used complex event processing to set rules for SOME/IP headers and communication behaviors. When behaviors violated the rules, they were defined as exceptions. The time and memory occupied by rule-checking were evaluated on an Intel Xeon E3-1275v3 CPU at 3.5 GHz and 16 GB of RAM. Tobias et al. [16] also designed a rule-based IDS. In addition, the rule table is digitally signed to prevent illegal tampering. This paper only makes a rough implementation and no details. Zhou et al. [41] designed a rule-based intrusion detection mechanism expressed in binary. Rules are converted to binary data according to a predefined definition and format. For example, 0x65, 0x02, 0x00, 0x03, 0x01, 0x22, 0x02, and 0x190 can be translated into the 65th rule, which is to alarm when the length of the payload segment of an Internet control message protocol (ICMP) packet is greater than 0x190. The author shows that the design can be adapted to the AUTOSAR system. However, the CPU utilization, memory usage, and ROM usage of IDS are tested only on a Raspberry Pi.

#### 2.2.2. AI-Based IDS

Seonghoon et al. [42] used a CNN method to detect anomalies in audio/video transport protocol (AVTP) streams. The author extracts the input features of the CNN network based on the observation of the payload segment. Experiments show that CNN can be used for anomaly detection in AVTP video streams, and the real-time performance of IDS is evaluated in Google Colab, Macintosh, Jetson TX2, and Raspberry Pi 3, respectively. The dataset used has been made public. Natasha et al. [43] verified the performance of a convolutional-based autoencoder (CAE), long short-term memory-based autoencoder (LSTM-AE), one-class SVM (OCSVM), local outlier factor (LOF), and isolation forest (IF) on the dataset of paper [42]. The author focuses on analyzing the real-time performance and model size of CAE and LSTM-AE. Alkhatib et al. [44] performed intrusion detection on SOME/IP packets using a sequence model. The detected dataset is generated by a SOME/IP generator [45]. The generator can only generate header data that conforms to the SOME/IP specification, and its payload segments are not related to each other. The detection focuses on SOME/IP communication behavior anomalies, such as an error on event/error and missing on response/request. The author compares the performance of a recurrent neural network (RNN) and LSTM without considering real-time requirements. Daniel et al. [46] proposed a hybrid intrusion detection architecture to detect Ethernet communications directly. The first layer is a static check, which detects obvious intrusions based on simple rule definitions, such as IP address tampering. At the same time, the static check will calculate some parameters as training features, such as the average time interval of each frame of packets, the entropy of the local IP address, etc. The authors evaluate the performance of three algorithms, principal component analysis (PCA), OCSVM, and Mahalanobis distance. However, the detection does not involve the payload segment.

### 2.3. Literature Comparison

From our research on the literature, most of the research on IDS of IVN focuses on the CAN bus, and the research on IDS for AE is still in its infancy. Only three of these studies are for SOME/IP, and there are obvious flaws. Although RNN is innovatively applied to IDS for SOME/IP in [44], the training or detection process lacks sufficient data and real-time performance analysis. As shown in the literature [16,40], the detection range only includes the header and process of SOME/IP.

Research gaps and problems can be summarized as follows: among the three pieces of research on SOME/IP IDS, they have yet to consider and realize the detection in payload, header, and communication process simultaneously. All the research experiments are insufficient, and the real-time performance analysis is lacking. To solve these problems, we propose a multi-layer IDS. The first layer adopts a rule-based detection method to detect headers and communication processes more efficiently. The second layer uses a multi-GRU model to detect anomalies in the payload. The detection and real-time performance are finally improved through parameter optimization. For the convenience of comparison, the advantages and differences between our paper and the other seven pieces of literature are listed in Table 1, where the symbol ✓ means to include.

## 3. Vulnerability of SOME/IP

SOME/IP is built on the TCP/UDP protocol and is located above the fourth layer of the OSI model. Its purpose is to define a unified middleware for IP-based communication within the vehicle. SOME/IP is one of the critical components to realizing the in-vehicle network communication under the service-based architecture. We first introduce the communication process of SOME/IP and clarify the application scenarios of Event and Remote Procedure Call (RPC) packets in SOME/IP. Then, we analyze its attack scenarios and attack types on SOME/IP protocol.

### 3.1. SOME/IP Overview

**Figure 1:**
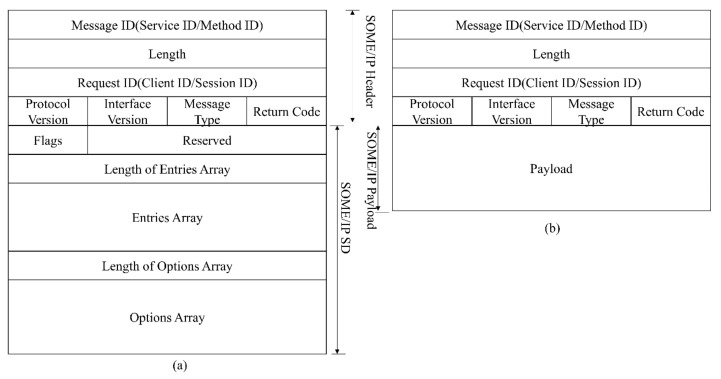
SOME/IP protocol format. (**a**) SOME/IP-SD message format. (**b**) SOME/IP message format.

The communication based on SOME/IP is divided into two phases. The first is the service discovery process, specified by SOME/IP Service Discovery Protocol [47], and the second is the normal communication process, specified by SOME/IP Protocol [48]. The SOME/IP-SD message and SOME/IP message format are shown in Figure 1.

The service discovery process is performed when the system starts, including three phases: initial wait, repetition, and main. Servers and clients notify each other of service information through SOME/IP-SD messages, consisting of the entries array and options array.

**Figure 2:**
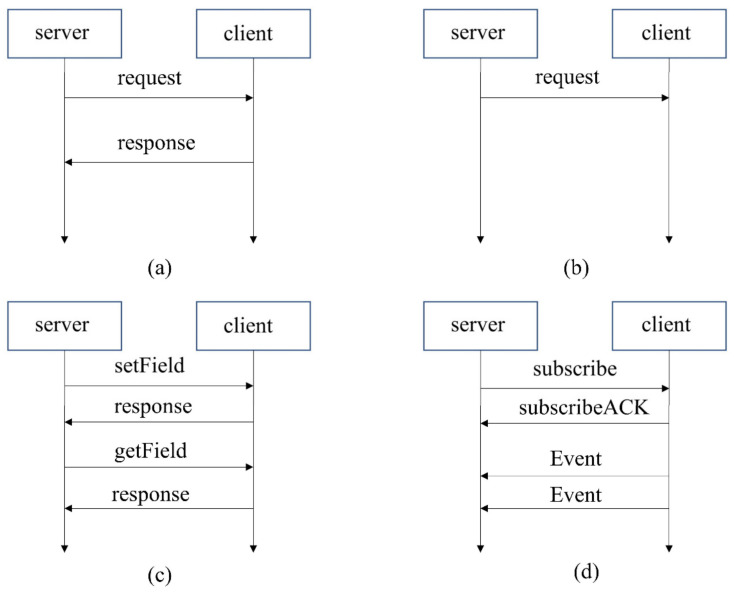
SOME/IP communication paradigm. (**a**) Request-Response-RPC. (**b**) Fire & Forget-RPC. (**c**) Setter & Getter of Field. (**d**) Publish-Subscribe for event.

A service consists of combinations of zero or multiple events, methods, and fields. Events provide data sent cyclically or on change from the provider to the subscriber. A field does represent the status and thus has a valid value at all times upon which the getter, setter, and notifier act. The communication of SOME/IP relies on RPC and Publish-Subscribe. RPC allows the client to call methods in the server. RPC contains two modes, Fire & Forget and Request-Response. The difference is that Fire & Forget does not need a response. Events in the service can only be transmitted after they have been subscribed. Operations of the field are special since the setter and getter of the field belong to Request-Response-RPC, while the notifier of the Field needs to be subscribed like an event. The communication paradigm of SOME/IP is shown in Figure 2.

In SOME/IP communication, events and RPC often act on different types of vehicle data. Since autonomous driving control algorithms require periodic and continuous inputs, the event is more suitable for real-time control and is primarily a fundamental signal. If the self-driving application uses RPC to trigger related calculation signals, it will increase the network load and reduce the real-time performance, resulting in a poor control effect. In contrast, RPC is more suitable for the interaction between humans and the vehicle or the control of body parts with low real-time requirements, such as calling the air conditioning control method or the turn signal control method through RPC.

### 3.2. Attack Scenario

**Figure 3:**
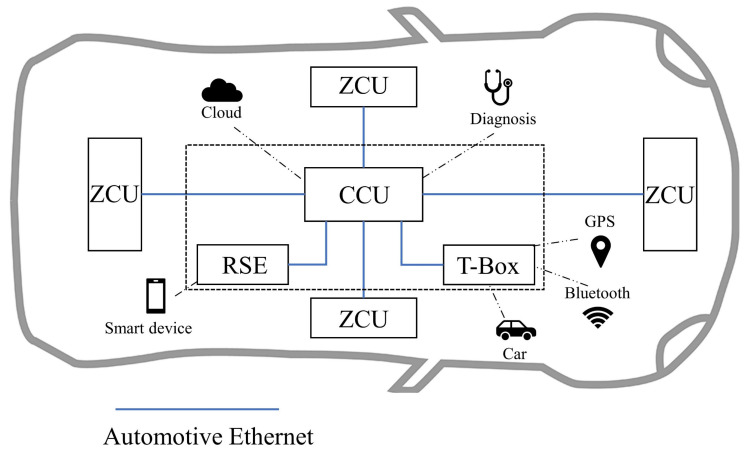
Automotive zonal EEA.

Figure 3 shows a zonal automotive electrical and electronic architecture (EEA). The automotive Ethernet is used as the backbone network to connect the zonal control unit (ZCU), central compute unit (CCU), rear seat entertainment (RSE), and telematics box (T-Box) in a star topology. SOME/IP runs as an upper-layer protocol in the backbone network. Four ZCUs are in charge of each of the four zones in the left, right, front, and rear of the vehicle. Each zone’s actuators, sensors, and sub-ECUs are connected to the ZCU via CAN or Ethernet. There are various external interfaces including Bluetooth, cellular network, GPS in RSE, CCU, and T-Box. Due to the need to perform diverse tasks such as information fusion, route planning, infotainment, etc., these electronic units are equipped with diverse operating systems such as Android, QNX, and Linux. An attack from the outside to the in-vehicle SOME/IP network is possible under the above EEA.

Due to the fixed topology of the AE-based IVN and the point-to-point communication method, it is almost impossible to attack the in-vehicle network by mounting malicious communication nodes directly, except in the ideal case. However, an attacker can infiltrate the SOME/IP network outside. A more feasible approach is that an attacker attacks straight from the data source, such as spoofing the camera, causing the speed sensor to have measurement errors, etc. In this scenario, all ECU nodes in the IVN are normal and communicate as expected. Moreover, there may be vulnerabilities in applications, operating systems, or virtual machines. It is possible to gain access to data or the network through these vulnerabilities or malicious software. For example, when an attacker obtains permission to operate the transmit interface of SOME/IP packet, attacks such as replay, tamper, fuzzy, and denial of service (DoS) can be launched on the network.

### 3.3. Attack of SOME/IP

#### 3.3.1. Fuzzy

Targets of fuzzy include the header of the event and RPC, service entries array, and option array in the service discovery packet. Fuzzy can also be understood as random or traversal tampering.

#### 3.3.2. Spoof

Spoof is considered an upgrade of Fuzzy. In our definition, the targets of Fuzzy do not contain the payload. The Fuzzy on the payload is invalid if the SOME/IP header does not match the requirements. Spoof means that the attacker can send the header of SOME/IP as required and tamper or replay the payload of the event at the same time. This requires a higher level of mastery of communication systems.

#### 3.3.3. DoS

DoS refers to the attacker congesting the network by modifying the cycle of periodic Events or SOME/IP-SD packets. DoS can also be achieved by injecting large amounts of traffic unrelated to SOME/IP. Nevertheless, this is not a SOME/IP level attack, and we can solve such DoS from the data link layer by introducing a flow meter or IEEE 802.1Qci [49].

#### 3.3.4. Abnormal Communication Process

The abnormal communication process mainly involves the four steps mentioned in the paper [44], including error on error, error on event, missing response, and missing request.

#### 3.3.5. Unauthorized Operation

Unauthorized operations do not exist in conventional CAN buses, which is mainly manifested in unauthorized subscription, unsubscription, provision of services, and unauthorized RPC calls. The services or RPCs here are defined in the system but have not been authorized by the upper application.

## 4. Proposed Multi-Layer IDS

### 4.1. Dataset Generation

**Figure 4:**
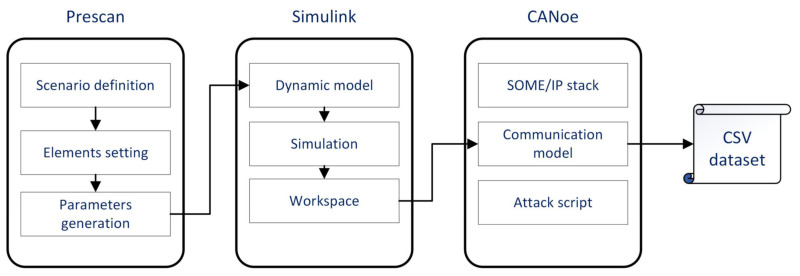
SOME/IP dataset generation process for IDS.

So far, there is no recognized SOME/IP dataset for intrusion detection in the industry. Since SOME/IP-based service-oriented communication has not been widely deployed in mass-produced vehicles, actual vehicle data communicating via SOME/IP cannot be collected. The literature [45] provides a SOME/IP data generator, but this generator can only generate the header of SOME/IP and fill in the payload with random numbers or fixed values. This makes it inconvenient to conduct a comprehensive intrusion detection study. The toolchain of Prescan, Simulink, and Vector CANoe is used to build the SOME/IP dataset to fill this gap. Prescan is a simulation platform for ADAS function development, which integrates modules involved in intelligent driving simulation, such as road scenes, smart cars, sensor models, vehicle dynamics configuration, and environmental perception. Simulink is a block diagram environment for modeling, analyzing, and simulating dynamic systems. CANoe is a bus development environment produced by VECTOR, which can be used for modeling, simulation, testing, and development of automotive buses. Simulating the ADAS function with Prescan and Simulink is one of the most common methods nowadays. Moreover, CANoe produced by Vector is widely used for in-vehicle network simulation and testing. Through this toolchain, traffic that meets the protocol requirements and has ADAS meaning can be generated. The data generation process is shown in Figure 4.

Prescan is first used to design and build vehicle simulation scenarios. Road elements such as vehicles and road signs can be added according to actual needs. A detailed set of parameters constrains each element. For example, the weight, wind resistance, running trajectory, dynamic parameters, etc., can be set for vehicle elements. The simulation scenarios and parameters defined in Prescan are then imported into the vehicle dynamics model and application sub-functions built in Simulink. Interrelated data in the simulation environment can be obtained, such as the sensor data, vehicle speed, throttle opening, hydraulic braking force, etc. The above data are then imported into CANoe and encapsulated by the SOME/IP protocol stacks implemented by the communication access programming language (CAPL). According to the defined service framework, these SOME/IP messages are transmitted between simulation nodes in CANoe. Finally, these messages are recorded through the logging file and form a CSV database through Python. The attack script is coded with CAPL and embedded in the emulation node. The attack can be executed via the panel, similar to the attack through the APP backdoor. It should be noted that this is not an actual attack scenario, but the same attack effect can be obtained.

### 4.2. System Structure

**Figure 5:**
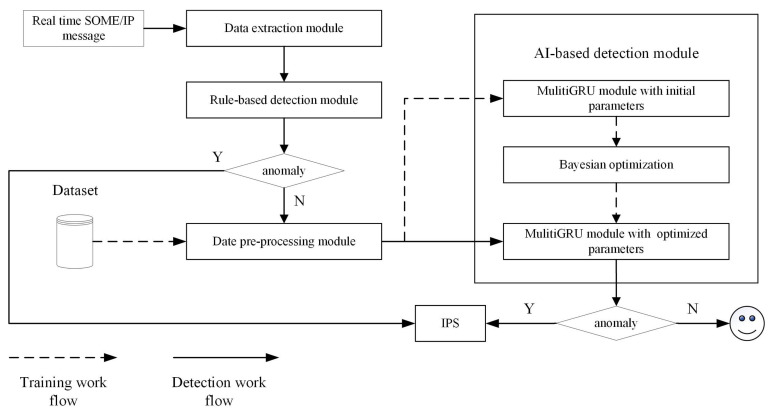
System architecture and workflow of multi-layer IDS.

The multi-layer IDS consists of rule-based detection and AI-based detection. The models in AI-based detection are trained before the IDS works appropriately. Figure 5 describes the system architecture of multi-layer IDS and reflects its workflow. In the training phase, the data are imported from the database into the data pre-processing module, which includes feature deserialization, normalization, and sequence generation. Sequence enters the initial multi-GRU model for training, and Bayesian optimization is used to obtain the hyperparameters of the model. During the detection phase, real-time SOME/IP packets flow from the IVN to the IDS. The packet enters the data extraction module, where the features for IDS are extracted. These features first enter the rule-based intrusion detection module. When all the rules are passed, the event packet will go through the pre-processing module to generate the feature sequence and enter the AI detection module. Other types of packets will jump out directly after entering the AI detection module and are marked as normal. When the results of both detection modules are normal, the packet is classified as normal. If any rules are not satisfied or the detection result of AI-based IDS is abnormal, the IPS will trigger related protection mechanisms, such as alerting, isolation, etc.

The detection range of our proposed multi-layer IDS list is outlined in Table 2. Except for unauthorized operations, the attacks mentioned in Section 3.3 can be detected by the proposed multi-layer IDS. For example, the driver can control the radio volume after the vehicle ignition, in which case an authorized RPC is generated. However, the attacker can issue an unauthorized RPC to control the radio volume anytime. The context of these two messages in the network is irregular. Such an attack cannot be identified at the network level if the service encapsulating RPC is offered. Hence, NIDS is only one part of the defense-in-depth system. Unauthorized operations must be defended by application probes, access control, or a host-based intrusion detection system (HIDS).

### 4.3. Data Extraction Module

Data extraction is a layer-by-layer unpacking process necessary for Ethernet communication. Each time the initial SOME/IP packet passes through a layer of the OSI model, the header of that layer will be added. After unpacking, the features for detection can be obtained.

### 4.4. Rule-Based Detection Module

All SOME/IP packets are first subjected to rule-based detection, which can detect anomalies in the SOME/IP-SD packet, SOME/IP header of event and RPC, and communication process. Each message ID, consisting of service ID and method ID, as shown in Figure 1, corresponds to a rule group. The rule group includes static field rules, dynamic field rules, and communication state rules. These rules will be judged in turn. As soon as a rule is not satisfied, an anomaly is flagged and pops up immediately. The packet is also marked as an anomaly if there is no corresponding message ID in the module. Parameters in the whole rule-based detection module are listed in Table 3.

The static fields mainly include IP Address, MAC address, port number, message ID, protocol version, interface version, message type, and information in the entries array and options array. These fields and their matching relationships are fixed after completing the service architecture and network topology design, such as the services the nodes can provide, the methods or event groups contained in the services, etc. Service description files can be extended through software-over-the-air (SOTA). In this case, static rules also need to be updated synchronously.

Dynamic fields refer to the timestamp of the SOME/IP packet and session ID in the SOME/IP header. The growth logic of session ID is defined in the specification [48] in detail. Attacks not complying with the session ID growth rules can be easily detected by checking this field, such as replay, tamper, and injection without the correct session id. Since SD and event packets are sent periodically, the injection of periodic packets can be detected by comparing the frame interval calculated by the timestamp with the set threshold.

The communication status rule detects the four process errors defined in the paper [44], error on error, error on event, missing response, and missing request. The rule-based module will cache the information of the previous packet. For example, after receiving a response-type SOME/IP packet, the module will check whether the previous frame of the same message ID is the request type.

### 4.5. Data Pre-Processing Module

#### 4.5.1. Deserialization and Data Restoration

Before filling the SOME/IP payload, the data will be serialized and converted to hexadecimal according to IEEE 754. A double-precision floating-point number takes 8 bytes. Both alignment and struct unwinding in serialization consume extra bytes. In order to reduce the feature dimension, the payload is converted into the real signal value through deserialization and data restoration.

#### 4.5.2. Data Normalization

Because the dimension of the actual signal is different, signals in each message ID are normalized separately, which can make the gradient descent process converge faster and improve training efficiency. At the same time, it is also avoided that the value range of different signals varies widely, resulting in poor model accuracy. The value of the normalized feature is denoted by:

(1) $$\;x_{n}=\frac{{x-x}_{\min}}{x_{\max}{-x}_{\min}}$$

where $x_{\max}$ is the maximum value of features and $x_{\min}$ is the minimum value of features.

### 4.6. AI-Based Detection Module Multi-GRU

#### 4.6.1. GRU

**Figure 6:**
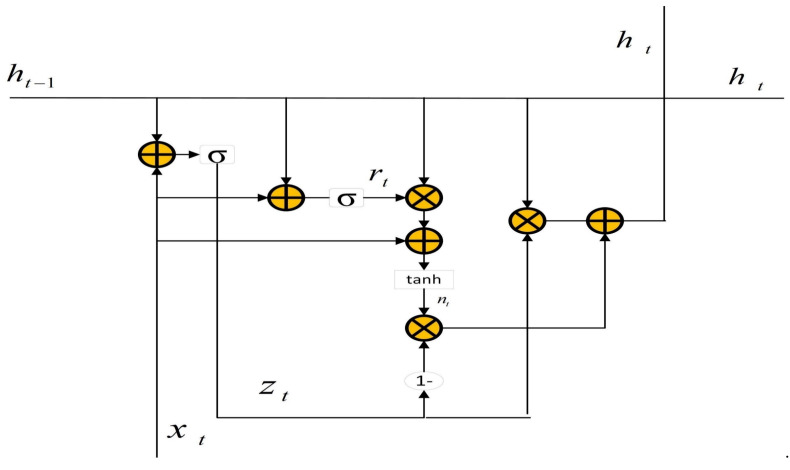
The principle of GRU.

GRU aims to solve the vanishing gradient problem, which comes with a standard recurrent neural network. The internal structure of GRU is shown in Figure 6. Compared with LSTM, GRU has only two gates, called the reset gate and the update gate. The update gate helps the model determine the amount of past information from previous time steps that must be passed along to the future. The reset gate decides the amount of past information to forget. The reset gate $r_{t}$ can be calculated by the following equation:

(2) $$r_{t}{=\sigma (W}_{\mathrm{ir}}x_{t}{+b}_{\mathrm{ir}}{+W}_{\mathrm{hr}}h_{t-1}{+b}_{\mathrm{hr}})$$

The update gate $z_{t}$ can be denoted by:

(3) $$z_{t}{=\sigma (W}_{\mathrm{iz}}x_{t}{+b}_{\mathrm{iz}}{+W}_{\mathrm{hz}}h_{t-1}{+b}_{\mathrm{hz}})$$

The candidate state $n_{t}$ can be expressed as:

(4) $$n_{t}{=\tan h(W}_{\mathrm{in}}x_{t}{+b}_{\mathrm{in}}{+r}_{t}{(W}_{\mathrm{hn}}h_{t-1}{+b}_{\mathrm{hn}}))$$

The hidden layer output $h_{t}$ is described as follows:

(5) $$h_{t}=\left({1-z}_{t}\right)n_{t}{+z}_{t}h_{t-1}$$

where $W_{\mathrm{ir}}$, $W_{\mathrm{iz}}$ and $W_{\mathrm{in}}$ are the weight matrices of input $x_{t}$; $W_{\mathrm{hr}}$, $W_{\mathrm{hz}}$ and $W_{\mathrm{hn}}$ are the weight matrices connected with the hidden layer output $h_{t-1}$; b is the bias.

#### 4.6.2. Architecture of Multi-GRU

**Figure 7:**
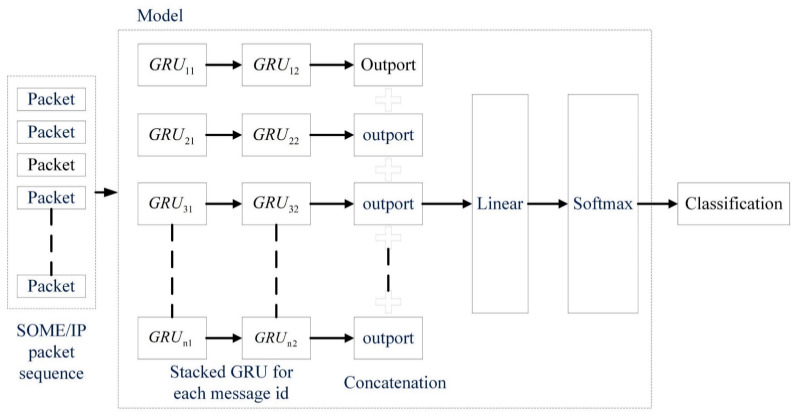
Structure of multi-GRU model.

Multi-GRU is a scalable supervised learning architecture with GRU as the core unit, as shown in Figure 7. Each message ID corresponds to a stacked GRU with a depth of 2. Increasing the network depth is intended to improve the efficiency and accuracy of model training and detection. The hidden layer outputs of all stacked GRUs are concatenated together to increase data interconnection between message ids. The classification results are obtained through the linear layer and the softmax activation function.

For a clear description, some symbols are first introduced, and the message ID is noted as id. ${\mathrm{ID}=\{\mathrm{id}}_{1}{,\;\mathrm{id}}_{2}{\ldots \mathrm{id}}_{n}\}$ is the set of all IDs. N is the number of elements in the ID, that is, the total number of IDs. Note the set X ${=\;\{x}_{\mathrm{id}1}{,x}_{\mathrm{id}2}{\ldots x}_{\mathrm{idn}}\}$, whose elements are the number of signals corresponding to the ID. Therefore, the total number of signals in the system is sum(X). Note that the set ${L=\{l}_{\mathrm{id}1}{,l}_{\mathrm{id}2}{\ldots l}_{\mathrm{idn}}\}$ contains the number of corresponding IDs in a sequence. Sum(L) represents the total length of a packet sequence. $h_{scale}$ is the hidden layer size for each signal. The parameters of the model are listed in Table 4.

**Figure 8:**
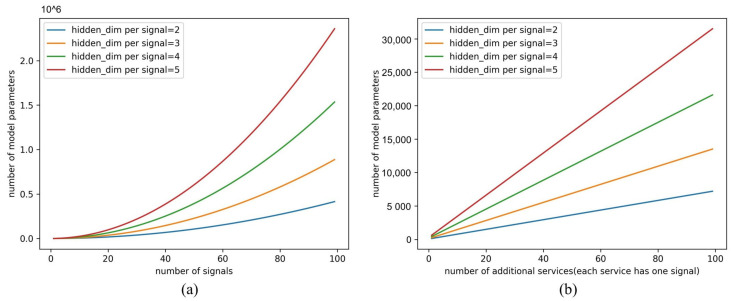
Number of model parameters with system expansion. (**a**) Number of parameters in single-GRU model with the increase in signal. (**b**) Number of parameters in multi-GRU model with the increase in service. One service corresponds to one signal.

Compared with the single-GRU model, the proposed multi-GRU model has better scalability. The number of IDs increases as the system expands. According to actual design requirements, the ID and signal may be 1-to-1 or 1-to-x. The extreme case is that all IDs have a 1-to-1 relationship with signals, which is also allowed and recommended for service-based communication. In the 1-to-1 case, the relationship between the number of parameters and N for the single-GRU and multi-GRU models is shown in Figure 8. When $h_{scale}$ is 5 and N reaches 40, the multi-GRU model has 12,000 model parameters, while the parameters of the single-GRU model are about 500,000, which is close to 40 times that of the former. The growth rate of single-GRU increases with N, which is a constant in the multi-GRU model.

#### 4.6.3. Model Hyperparameters

Adam is used for optimizer and cross-entropy for the loss function. Adam absorbs the advantages of Adagrad (adaptive learning rate gradient descent algorithm) and momentum gradient descent algorithm, which can adapt to sparse gradients and alleviate the problem of gradient oscillation. Hence, the model hyperparameters are the hidden layer size $h_{scale}$, the learning rate lr, and the smoothing parameters β1 and β2 of the Adam optimizer. If the hyperparameters are not correctly chosen, the training process will be unstable and ineffective. Manual selection of hyperparameters is inefficient and has difficulty with obtaining optimal solutions, so Bayesian optimization is used to calculate hyperparameters automatically. Bayesian optimization uses Bayes’ theorem to estimate the posterior distribution of the objective function based on the data and then selects the next sampled hyperparameter combination based on the distribution. It makes full use of the information from the previous sampling point. Its optimization works by learning the shape of the objective function and finding the parameters that improve the result to the global maximum. The Bayesian optimization objective is the model accuracy of threefold cross-validation. The optimized hyperparameters and ranges are shown in Table 5.

**Figure 9:**
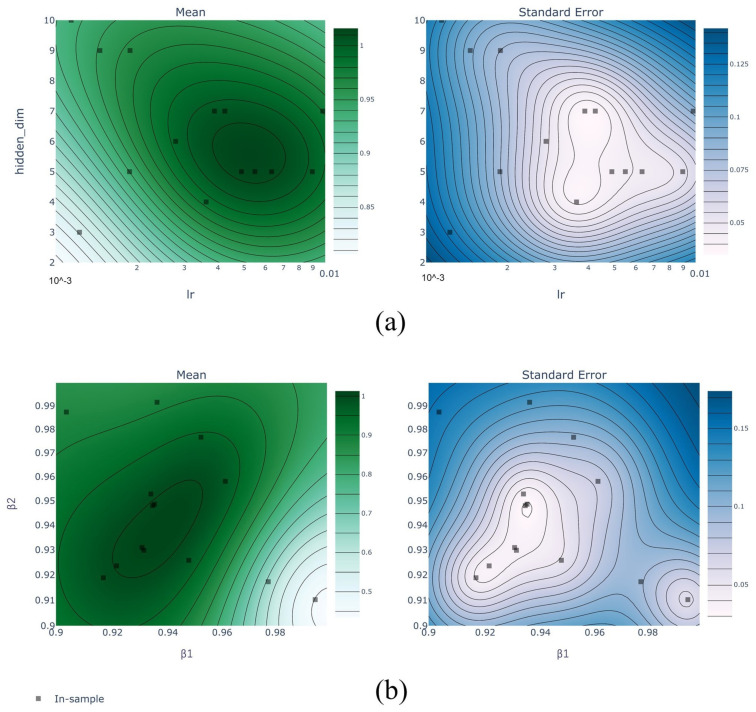
Bayesian optimization process with hyperparameters. (**a**) Contour distribution of mean and standard error of threefold cross-validation accuracy with *h_scale_* and lr. (**b**) Contour distribution of that with β1 and β2.

The mean and standard error of the threefold cross-validation accuracy under different hyperparameters are shown in Figure 9. This figure intuitively shows the influence of hyperparameters on the accuracy of the model. The closer the distribution of error and standard error are to the center of the contour, the better the hyperparameters are.

## 5. Performance Evaluation

### 5.1. Data Description

The dataset was generated by the method described in Section 4.1. The simulation environment is an adaptive cruise control (ACC) scenario, and the service and signal definitions are shown in Table 6. The dataset contains the SOME/IP-SD packet, four events, and two RPCs. The event is sent periodically, and RPC is triggered via the CANoe panel.

The dataset is divided into two parts for validating rule-based and AI-based detection. The full dataset and its detailed description are available in git [50]. For the evaluation of rule-based detection, there are 144,574 packets in total, of which 89,564 are anomalies. Details are shown in Table 7.

There are 2480172 original data samples in the evaluation of AI-based detection. A total of 82625 message sequences are obtained with a sequence length of 91 and a sliding step of 30. At the beginning of our experiments, we found that a low sequence length leads to a low replay attack detection rate. After coarse-grained tuning, the sequence length was determined to be 91, seven times the number of messages in one communication cycle in the dataset. The sliding step was empirically determined to be 30% of the sequence length. If the sliding step is too small, too much redundant information will be generated, leading to over-fitting of the training. If the sliding step is too long, it will lead to the omission of crucial information and a reduction in the amount of data. We use an 80–20% train-test split to generate a training set with 80% of data samples and a test set with 20% of data samples. The test set will remain untouched before the final hold-out validation. The class label and size of the dataset for AI-based detection are shown in Table 8. It should be pointed out that the header and cycle of tamper and replay attacks meet the system requirements. We can assume that the attackers have successfully spoofed the rule-based IDS and impersonated the nodes loaded with these services. So, these attacks will focus on the payload.

### 5.2. Experiment Setup

The development platform is a laptop with an IntelI CITM) i7-8750H CPU @ 2.20 GHz and 16 G of memory. The detection performance and computational performance of multi-GRU and single-GRU are verified and compared on this platform. The experimental platform is a Jetson Xavier NX with a 384-core NVIDIA Volta^TM^ GPU, two deep learning acceleration engines, a 6-core NVIDA Carmel ARMv8.2 64-bit CPU, and 8G of memory. The Jetson Xavier NX is an embedded edge computing device with only 15 W power consumption and 21 TOPS of computing power. With only the CPU, its arithmetic power is roughly the same as that of the Raspberry Pi 4B. On this platform, we perform a vehicle-level real-time analysis of the proposed multi-layer IDS, including the detection time of the rule-based module and the detection time of the AI-based module with CPU or GPU acceleration, respectively.

### 5.3. Evaluation for Rule-Based Detection

The experiments show that the proposed rule-based detection has a 100% detection rate in the above dataset. This result is understandable because rule-based judgments are rigorous. Real-time performance is another primary metric in rule-based detection. This paper does not store the rules in the rule base but deploys them in the software code in the form of logical judgment. Such an implementation will not consume additional memory of a rule base, nor will it affect the real-time performance due to the retrieval of the rules, which is more suitable for the embedded environment. Due to positive logical judgments (as a whitelist), packets that do not meet the conditions are immediately considered abnormal. Therefore, it takes the longest time to judge a normal packet, and this time is used to evaluate its time performance. The average detection time of each packet is about 29.394 us on the Jetson Xavier NX. After the packet enters the system, it will enter the corresponding rule set according to the message id. Thus, its inference time hardly increases with the system’s expansion.

### 5.4. Evaluation for AI-Based Detection

In addition to real-time performance, there are some other metrics in the evaluation of AI-based detection, including the accuracy (Acc), precision, recall, and F1-score, which are calculated as follows.

(6) $$\mathrm{Acc}=\frac{\mathrm{TP}+\mathrm{TN}}{\mathrm{TP}+\mathrm{TN}+\mathrm{FP}+\mathrm{FN}}$$

(7) $$\;\mathrm{Recall}=\frac{\mathrm{TP}}{\mathrm{TP}+\mathrm{FN}}$$

(8) $$\mathrm{Precision}=\frac{\mathrm{TP}}{\mathrm{TP}+\mathrm{FP}}$$

(9) $$F1=\frac{2\times \mathrm{Precision}\times \mathrm{Recall}}{\mathrm{Precision}+\mathrm{Recall}}$$

where TP, TN, FP, and FN represent true positives, true negatives, false positives, and false negatives. The area under the ROC curve (AUC) was also used to judge the model’s classification accuracy. The performance of the multi-GRU model is compared with that of a single-GRU to illustrate the advantage of the proposed model. The hyperparameters of both models were tuned by the Bayesian process for a better comparison and are listed in Table 9.

**Figure 10:**
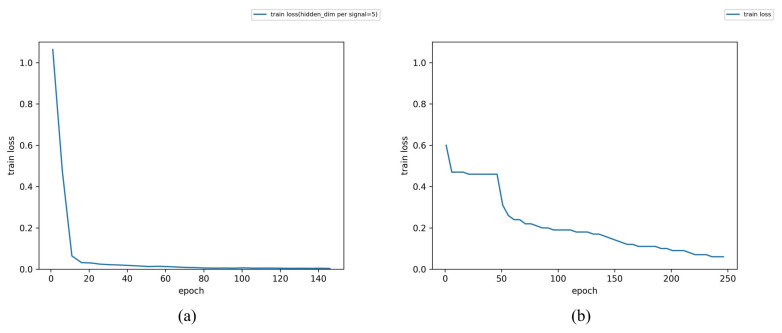
Train loss of models. (**a**) Train loss of multi-GRU model. (**b**) Train loss of single-GRU model.

**Figure 11:**
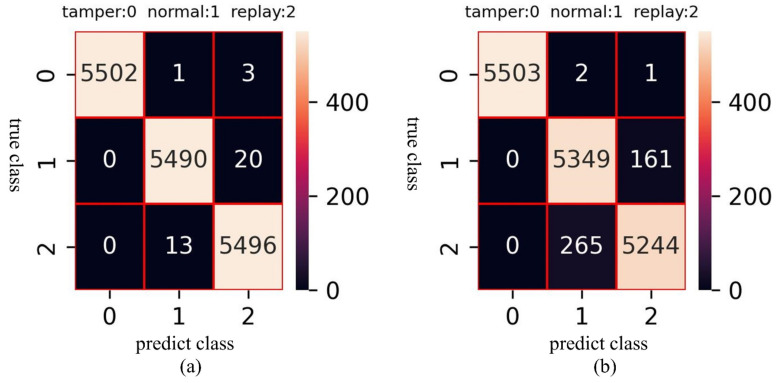
Confusion matrix of models. (**a**) Confusion matrix of multi-GRU. (**b**) Confusion matrix of single-GRU.

In Figure 10, it is evident that the loss of the multi-GRU model converges faster and closer to zero during training. The training loss is close to 0 in less than 60 epochs in multi-GRU training. On the contrary, after 250 epochs in single-GRU training, the training loss is still a little far from 0. The detection performance of the two models against different attack types is listed in Table 10. It can be found that the multi-GRU model has excellent detection performance for tamper, normal, and replay, and the accuracy is as high as 99.77%. In contrast, although the single-GRU model also has a very high detection rate for tamper data, its detection performance in the replay and normal data is poor. The accuracy of this model is 97.4039. A more intuitive classification result can be shown in Figure 11. It can be found that the single-GRU model has serious misjudgments between replay and normal data.

Table 11 compares the computational performance of the two models on a laptop. The detection time of multi-GRU for a sequence is 21.5838 ms, which is nearly 10 ms less than that of single-GRU. Since the evaluation data set contains only one ACC application and only four message IDs of the event, the number of model parameters of single-GRU is slightly less than that of multi-GRU. However, according to the scalability analysis in Section 4.6.2, as the system expands, the number of model parameters of multi-GRU will far exceed that of single-GRU. Floating-point operations per second (Flops) can also demonstrate the advantages of multi-GRU in computing performance. The Flops of single-GRU are quintuple that of multi-GRU.

It is noted that the inference time includes the data pre-processing time and model calculation time. We compared the composition of the inference time of multi-GRU using a Jetson Xavier NX with CPU or GPU acceleration, as shown in Table 12. The GPU quadruples the model computation speed but has little effect on the speed of data pre-processing. This result demonstrates the computational potential of the proposed model under GPU acceleration. Data pre-processing time can be further reduced by dedicated chips or more efficient data processing algorithms, but this is not the focus of this paper. The inference time for the Jetson Xavier NX is less than 1 ms regardless of whether GPU acceleration is used.

### 5.5. Performance of Resistance to Sample Imbalance

In the actual vehicle scene, sample imbalance often occurs. The performance of the model under unbalanced samples is also an important indicator. Mild, moderate, and extreme sample imbalance scenarios are tested, where the ratio of abnormal data to normal data, denoted as the ratio later, is 40%, 20%, and 1%, respectively. Table 13 shows the performance of the proposed multi-GRU model and the traditional single-GRU model for the above scenarios. The balanced test dataset is used so that the performance of the model trained in the unbalanced sample environment is better represented. Generally, the proposed multi-GRU model has a solid resistance to sample imbalance. At ratios of 40% and 20%, the recall of replay attacks can still be maintained at about 90%. In contrast, the replay attack is already undetectable at a ratio of 40% under a single-GRU model. The training of the single-GRU model fails to converge under sample imbalance, and the loss fluctuates between 0.3 and 0.4. In the case of extreme sample imbalance (ratio = 1%), the multi-GRU model also exhibits poor performance, which is unavoidable because the number of negative samples is too small to extract enough features during the learning process. For this case, it is necessary to use oversampling, weight distribution, or data generation algorithms to balance the samples, which is not the research content of this paper. With the dataset generation method described in Section 4.1, we can set the experimental scenario and attack injection frequency to adjust the total number of samples and the ratio of abnormal data to normal data.

### 5.6. Vehicle-Level Real-Time Analysis

In the IEEE 802.1DG seminar, the in-vehicle network traffic and its real-time requirements are defined in Table 14.

The time of rule-based detection is only 29.394 us per packet for the Jetson Xavier NX, which fully meets the vehicle-level real-time performance requirements. However, event pavoltckets must go through two layers of the proposed IDS simultaneously. Therefore, the detection time of an event is the sum of rule-based detection and AI-based detection. In NX, the average detection time of each event packet is 0.3958 ms with GPU acceleration and 0.6669 ms with only the CPU. In terms of the detection time of a single packet, this also meets the real-time requirement. Nevertheless, it should be pointed out that the detection unit of RNN is a sequence. It is technically challenging to achieve real-time detection unless the sequence sliding step is 1. We believe that the number of packets inferred per unit time is also a metric for evaluating real-time performance. If this metric exceeds the number of packets appearing in the network per unit time, undetected packets are not continuously accumulated, resulting in an untimely response.

Our proposed multi-layer IDS can process 2526 event packets per second with GPU acceleration and 1499 with only a CPU converted from the above statistics. Since there are no real vehicle SOME/IP data, it is impossible to give a quantitative analysis of the real-time performance of the real vehicle. As described in Section 3.1, a periodic event is mainly used for real-time control. The variety of real-time control messages is limited and mainly comes from the base services in the chassis, power, and ADAS domains. It can be predicted that there will be no more than 40 real-time control signals in the IVN, such as braking force, throttle opening, data processed by sensors, etc. Since the automotive Ethernet is a point-to-point connection, the arrangement of the IDS determines the amount of traffic flowing through it. If the distributed arrangement is adopted, the number of events in a single link is roughly consistent with the simulation data in this paper (13 signals, 325 event packets per second), so the proposed multi-layer IDS can meet the real-time requirements. If the proposed multi-layer IDS is deployed at a central gateway or central calculation unit to form a centralized IDS, the number of model parameters in AI-based detection increases due to more signals. The rising metric and declining computing performance may prevent the proposed multi-layer IDS with only the CPU from meeting the real-time requirement. To solve this problem, we can also select the more critical traffic in the network to be detected during the centralized arrangement or use GPU acceleration.

However, whether using a CPU or GPU acceleration, not all anomalous threat detection has the highest priority. This means that resources inevitably need to be prioritized in resource-constrained vehicle scenarios to meet safety-related operations of IoV, such as GPU requirements for ADAS or autonomous driving applications. The impact of different batch sizes on GPU acceleration performance is shown in Table 15. Received data can be stored when GPU resources are temporarily constrained. We can detect large batches of data when the GPU is idle or periodically to achieve time-sharing utilization of the GPU and higher GPU efficiency. The batch size needs to be chosen according to the actual resource usage.

## 6. Conclusions

This paper proposes a multi-layer intrusion detection system for SOME/IP-based in-vehicle network communication. The first-layer rule-based detection module is mainly for SOME/IP-SD and SOME/IP header Fuzzy, DoS, and the abnormal communication process. The average detection time of each packet for a Jetson Xavier NX is 29.394 us. The second-layer AI-based detection module is mainly used to detect spoof attacks on the event. In this module, this paper proposes the model structure of multi-GRU, and experiments show that this structure has higher detection accuracy and computational efficiency than the single-GRU model. The AI-based detection module with multi-GRU has a detection accuracy of 99.7761% for spoof attacks and can distinguish the type of attack on the payload, 100% for Tamper, 99.7456 for Normal, and 99.5833 for Replay. The total time consumption of the multi-layer IDS for the Jetson Xavier NX is 0.3958 ms with GPU acceleration and 0.6669 ms with only the CPU.

After vehicle-level real-time analysis, our proposed multi-layer IDS has better computational potential with GPU acceleration and is more suitable for distributed layout or only to select critical ADAS traffic for detection in a centralized layout. This paper also proposes a SOME/IP dataset establishment method to make up for the deficiencies in the dataset of SOME/IP.

In future work, we need to further investigate the GPU acceleration policy in resource-constrained environments and expand the dataset to verify the performance of the proposed IDS under large-scale networks or on real car data if possible. The HIDS also needs to be studied to address unauthorized calls in SOME/IP.

## Figures and Tables

**Table 1:** Comparation of literature on intrusion detection on automotive Ethernet.

Paper	AI-Based	Rule-Based	SOME/IP	Model Innovation	Real-Time Consideration	Detection in Header/Process	Detection in Payload
Seonghoon et al. [42]	✓				✓		✓
Natasha et al. [43]	✓				✓		✓
Alkhatib et al. [44]	✓		✓			✓	
Nadine et al. [40]		✓	✓		✓	✓	
Tobias et al. [16]		✓	✓			✓	
Daniel et al. [46]	✓	✓				✓	
Zhou et al. [41]		✓				✓	
Our	✓	✓	✓	✓	✓	✓	✓

**Table 2:** The detection range of multi-layer IDS.

Attack Type	Detection Module	Content
Fuzzy	Rule-based	Header of event, RPC and SOME/IP-SD packet; service entries array and options array in SOME/IP-SD packet
Spoof	AI-based	Payload of SOME/IP event
DoS	Rule-based	Interval of event and SOME/IP-SD packet
Abnormal communication process	Rule-based	Communication process

**Table 3:** Fields used in rule-based detection module.

Field Name	Description
IP Address	Static field in the header of SOME/IP and SOME/IP-SD packet
MAC address	
Port number	
Message ID	
Protocol version	
Interface version	
Message type	
Client ID	
Find service entries array	
Offer service entries array	
Eventgroup array	
Options array	
Session ID	Dynamic field
Interval of packet	
Status cache of the previous message	Communication status parameter
The estimated status of the next message	

**Table 4:** The architecture of multi-GRU.

Multi-GRU Architecture	
Layer	Output Dimension
First stacked GRU per ID	[$l_{\mathrm{idn}}$, $x_{\mathrm{idn}\;}\times \;h_{scale}$]
Second stacked GRU per ID	[1, $x_{\mathrm{idn}\;}\times \;h_{scale}$]
Concatenation	[1,$\;\mathrm{sum}(X)\;\times \;h_{scale}$]
Linear	[1,3]

**Table 5:** Optimized range of hyperparameters.

Hyperparameter	Range
$h_{scale}$	[2, 10]
Lr	[0.001, 0.01]
β1	[0.9, 0.9999]
β2	[0.9, 0.9999]

**Table 6:** Services and signals in the dataset.

Message ID	Number of Signals	Type	Signal Description
0x14720011	2	Event	Brake pressure and throttle opening
0x14720012	2	Event	Preceding vehicle speed and collision warning time
0x27590010	6	Event	Distance, doppler velocity and degree relative to preceding vehicle from sensor 1 and sensor 2.
0x36120009	3	Event	Velocity, heading and y-axis rotation angle of the vehicle
0x15880008	1	Request-Response-RPC	Set air conditioning temperature
0x15880007	1	Fire & Forget-RPC	Turn on the air conditioning

**Table 7:** Dataset for rule-based detection.

Total	Normal	Fuzzy	DoS	Abnormal Communication Process
144,574	55,010	43,867	12,188	33,509

**Table 8:** Class label and size of dataset for AI-based detection.

Attack Type	Class Label (Value)	Train	Test
Spoof	Normal (1)	22,025	5506
	Tamper (0)	22,037	5510
	Replay (2)	22,038	5509

**Table 9:** Hyperparameters of two models for performance evaluation.

Model Type	$h_{scale}$	lr	β1	β2
Multi-GRU	5	0.0089630704	0.933792409392	0.952802490181
single-GRU	31	0.0043259137	0.939844012507	0.943045819607

**Table 10:** Performance evaluation of two models on dataset.

	Attack Type	Precision (%)	Recall (%)	F1-score	AUC	Accuracy (%)
Multi-GRU	Tamper	100	99.9274	0.9996	0.9996	99.7761
	Normal	99.7456	99.6370	0.9969	0.9975	
	Replay	99.5833	99.7640	0.9967	0.9978	
Single-GRU	Tamper	100	99.9455	0.9997	0.9997	97.4039
	Normal	95.2457	97.0780	0.9615	0.9733	
	Replay	97.00	95.19	0.9609	0.9686	

**Table 11:** Calculation performance comparison of two models in laptop.

	Multi-GRU	Single-GRU
Inference time per sequence (ms)	21.5838	31.7356
Inference time per message (ms)	0.2372	0.3488
Number of model parameters	13,698	10,047
Flops	205,855	945,128

**Table 12:** Real-time analysis of multi-GRU using a Jetson Xavier NX.

Multi-GRU	Time Consumption with Only the CPU (ms/packet)	Time Consumption with GPU Acceleration (ms/packet)
Data pre-processing	0.2994	0.2841
Model calculation	0.3381	0.0823
Inference time in total	0.6375	0.3664

**Table 13:** Model performance comparison under sample imbalance.

	Attack Type	Precision (%)	Recall (%)	F1-Score	Accuracy (%)
Ratio = 40%					
Multi-GRU	Tamper	99.9816	98.8921	0.9943	97.7610
	Normal	94.0613	99.7459	0.9682	
	Replay	99.5798	94.6451	0.9705	
Single-GRU	Tamper	100	99.7821	0.9989	66.5779
	Normal	49.9454	99.6189	0.6653	
	Replay	46.3415	0.3449	0.0068	
Ratio = 20%					
Multi-GRU	Tamper	99.7245	98.6197	0.9916	96.6657
	Normal	91.2481	99.9093	0.9538	
	Replay	99.8415	91.4685	0.9547	
Single-GRU	Tamper	100	99.8365	0.9992	66.7897
	Normal	50.1025	97.6225	0.6622	
	Replay	55.1370	2.9225	0.0555	
Ratio = 1%					
Multi-GRU	Tamper	100	81.5292	0.8982	61.0287
	Normal	46.2431	99.8548	0.6321	
	Replay	68.1159	1.7063	0.0333	
Single-GRU	Tamper	100	97.5118	0.9874	65.8336
	Normal	49.3905	100	0.6612	
	Replay	0	0	0	

**Table 14:** Real-time requirement of in-vehicle traffic.

Traffic Type	Latency (ms)
Safety-relevant control	<1
Safety-relevant media	<1
Network control	None
Safety-irrelevant control	<50
Safety-irrelevant media	<300
Best effort	None

**Table 15:** Detection time consumption of GPU with different batch sizes.

Batch Size	Time Consumption of Model Calculation with GPU Acceleration (ms/packet)
1	0.0823
5	0.0445
10	0.0275
50	0.0180
100	0.0161

## Data Availability

The dataset has been uploaded to github [50].
